# Powers of County Medical Societies

**Published:** 1857-08

**Authors:** 


### ART. IV.—*Powers of County Medical Societies. John D. Hill vs. The Erie County Medical Society.*

*Supreme Court.*—The People *ex. rel.* John D. Hill *vs.* The Erie County Medical Society.

This cause was argued at the May Special Term. Sherman S. Rogers, Counsel for Relator; and Isaac W. Thompson, Counsel for the Defendant.

An alternative mandamus had been issued in the case, to which the defendant made return.

The cause came up for argument upon demurrer to a replication interposed to the relator's plea.

The object of the proceeding is to compel the defendant to restore the relator to the enjoyment of his privileges as a member of the society, from which he had been expelled because of an alleged infraction of the tariff of prices adopted by that society. That infraction consisted, as was claimed by the defendant, in taking the office of physician to the Alms' House of Erie County, for a less sum than the minimum price fixed by the tariff.

The nature of that tariff, and the history of its adoption, are set forth in the opinion of the court in the case of The People, *ex. rel.* Grey, in which case most of the questions here arising were presented.

It was claimed, however, in this case, that the relator never was a member of the society, he not having filed a copy of his diploma with the Clerk of Erie County at the time he made application for membership, and not having signed the laws of the society. It appeared from the pleading that, notwithstanding his failure to do these things, he had been for several years acting as a member of the society; had been an officer thereof; paid dues, &c., and in all respects been recognized and treated by the society as a member, until after his alleged illegal expulsion for non-compliance with the tariff of prices.

The case was decided yesterday,—the court maintaining the demurrer, and holding that the relator was entitled to a restoration of all the privileges as a member of the society.

The following are the conclusions arrived at by the Court, Marvin J.:

1. "Dr. Hill was a lawful member of the society notwithstanding he had not filed his license with the county clerk, and did not sign the by-laws. He was recognized for years as a member, and enjoyed all the rights and privileges of a member. He was expelled for the reason that he did not conform to the regulation of the society touching the tariff for services. The society cannot now change its ground, or resort to the objections, highly technical, that he had not filed his license, or signed the by-laws.

2. "The relator was illegally expelled for the reasons stated in my opinion in the case of The People *ex. rel.* Gray.

3. "The issues of the fact joined are not material, and it cannot be necessary to send them to a trial by jury. Upon the conceded facts in the case the relator is entitled to the relief he asks. There must be a peremptory mandamus."

At the same term at which this cause was argued, a motion *ex parte* Eldred P. Gray, was made upon which the court took the papers, and yesterday upon granting the motion, read the following opinion:

The relator represented that he had been duly licensed to practice as a physician and surgeon in this state.

That at a regular meeting of the Medical Society, in the County of Erie, held in January, 1850, he was duly admitted a member of the society.

That at a semi-annual meeting of the society held in June, 1854, a tariff of fees for medical services and medical appointments in and for the city of Buffalo and Erie county, was adopted in the absence of the relator, viz:

For Physician to the Alms-house of the county, $6.00 as the minimum price.

For Physician to the Penitentiary of the county, $3.00 as the minimum price.

For Physician to the Jail of the county, $1.50 as the minimum price.

For Physician to the Poor of the city of Buffalo, for Medical District, No. 1, $2.50; District No. 2, $1.25; District No. 3, $1.25; District No. 4, $1.00.

For attending Coroner's Inquests, post-mortem examinations, $10, and for viewing body, $5; mileage to be charged according to the rates of the Buffalo fee-bill.

For Health Physician of the city of Buffalo, $1000 as the minimum price.

The society at the same meeting, also adopted a resolution that it should be dishonorable for any member of the society to accept any appointment or perform any service contained in such tariff of prices, at a less sum than therein specified.

In October, 1855, the relator was appointed by the Board of Supervisors of the county of Erie, to visit the jail and perform the medical services, that were required during the year then ensuing at and for the compensation of one dollar a visit. The relator accepted the appointment and entered into an agreement with the Board of Supervisors, and entered upon the performance of the agreement.

At the semi-annual meeting of the Medical Society, certain proceedings were had in relation to the relator, and a committee was appointed to report upon the subject of complaint.

The committee reported among other things, that the relator had been guilty of an infraction of the laws of the society, and reported certain resolutions which were adopted by the society.

1. That agreeing to serve the public authorities for any other minimum amount and by any other manner of compensation than by salary, as fixed by the rules of the society is a clear and palpable infraction of its laws.

2. That the action of Dr. Gray in accepting office as admitted by himself before the society this day, merits and hereby receives an expression of the disapprobation of the society; and that provided he immediately retires from the position which he holds in violation of the rules of the society, further action in the case shall be waived.

A copy of these resolutions was served upon the relator, who declined to retire from the position he occupied under his contract with the Board of Supervisors. At an adjourned meeting of the society, the relator was, by a vote of the members of the society, expelled from the society for the cause here stated.

At a subsequent meeting, the society refused to recognize the relator as a member of the society, and to receive his vote at an election of officers of the society.

The affidavit of the relator contained a very full and minute statement of the facts relating to the question of his rights, and his expulsion from membership, but the facts as here briefly stated, are sufficient to present the real question, and the question discussed by the courts.

James G. Hoyt, for Relator.

Marvin, J.—The motion must be granted. It is impossible in any view which I have been able to take of the question presented by this motion, to sustain the action and position of the "Medical Society of the County of Erie." The society has mistaken its rights and powers. It will be important to ascertain what this corporation is; the object of its creation and the powers with which it is clothed; so far at least, as to determine whether it was vested with the power exercised in the present case.

The corporation has its existence under the act "to incorporate Medical Societies *for the purpose of regulating the practice* of physic and surgery in this State," passed April 10, 1813. The practice of physic and surgery in the city of New York, was regulated by law as early as 1760, and general regulations for the whole State were adopted in 1796, by which the Chancellor, a Judge of the Supreme Court or Common Pleas, or a Master in Chancery, were authorized to license physicians and surgeons to practice. In 1806, an act was passed establishing Medical Societies in the State, and a State Medical Society, and repealing the former act. The act of 1813 was a revision of the act of 1806.

It is important to notice the preamble to the act of 1813, that "Whereas well regulated Medical Societies have been found to contribute to the diffusion of that science, and particularly the knowledge of the healing art."

The first section makes it lawful for the physicians and surgeons in the several counties of the State, who were then authorized by law to practice in their several professions, except in those counties where medical societies had been incorporated, to meet together and choose the officers named in the act, and upon being so organized it was declared that such societies should be bodies corporate and politic.

The Medical Societies of counties already incorporated were to continue to be bodies corporate and politic.

The act contains many provisious touching the meetings and proceedings of the societies.

They are authorized to examine students, and for this purpose to appoint censors and to give diplomas.

By the 13th Section, the societies are authorized to make such by-laws and regulations relative to the affairs, concerns, and property of said societies; relative to the admission and expulsion of members; relative to such donations or contributions as a majority of the members at their annual meeting shall think fit and proper: provided that such by-laws, rules, and regulations be not contrary to, nor inconsistent with, the constitution and laws of this State, &c.

The power is here given to make by-laws and regulations relative to the *admission and expulsion of members.* It is in general terms. But this is not an arbitrary, unlimited power. The by-laws, rules and regulations are not to be contrary to nor inconsistent with the laws of the State.

Regarding the tariff of prices for medical services adopted at the semi-annual meeting in June, 1854, as a regulation, the question will arise whether it was a valid regulation authorized by law; and whether for a violation of it by a member, the society had the power of expulsion.

A serious doubt may be here raised, whether, if the matter of the regulations is not objectionable it ever had any binding force.

This regulation was made at a semi-annual meeting of the corporation The statute only authorized the society to make by-laws and regulations at its *Annual Meeting.* It may be very important that the power to make by-laws, rules and regulations, for a violation of which penalties upon members may be imposed, should only be exercised at the *Annual Meeting*, or at certain times fixed by law, so that all the members may know in advance and attend. But I shall waive this question, and proceed at once to the important question in the case, whether the society had the authority to establish such regulations, and for a breach of it to expel the relator.

It is declared by one of the by-laws of the society, that every member who shall neglect or refuse to comply with the by-laws and regulations of this society, &c., &c., shall be expelled from said society upon a vote of a majority of the members present. This can, of course have no application except to a neglect or refusal to comply with such by-laws and regulations as the society was authorized by law to make, and also to enforce by expulsion.

When a corporation is duly created the law tacitly annexes to it the power to make by-laws or private statutes for its government and support, so that this corporation in the present case, would have had the power to make by-laws had the statute been silent upon that question.

It is usual to confer the power by the charter or law authorizing the corporation.

If the power is expressly conferred and in general terms, it is construed as an authority conferred for the purpose of enabling the corporation to accomplish the objects of its creation, and the power in its exercise, is to be limited to such objects or purposes. Ang. & Ames on Cor. 268, 2d Kent's Com. 296. Grant on Cor. 76.

A by-law must not be at variance with the general law of the land. It must be reasonable and adapted to the purposes of the corporation. Kent says these corporate powers of legislation must be exercised reasonably and in sound discretion, and strictly within the limits of the charter, and in perfect subordination to the constitution and general law of the land and the rights dependent thereon. Subject to these limitations, the power to make by-laws may be sustained and enforced by just and competent pecuniary penalties. 2d Kent Com. 296. Grant on Cor. 76. Ang. & Ames, 275, 281, 286. Rex *vs* Tho. Cooper, of Newcastle, 7, 1, R. 548. 3d East. 186.

What were the objects and purposes of this corporation? In the preamble to the act, the legislature say: that well regulated Medical Societies have been found to contribute to the *diffusion of true science, and particularly the knowledge of the healing art.* The act then authorized the physicians and surgeons in the several counties of the State, to meet and choose certain officers, and when so organized declared them to be bodies corporate and politic. It provides for the examination and licensing of students, and for the mode of managing its affairs. Can it be said with any plausibility, that the establishing of a tariff of prices for medical services, was a legitimate object of the creation of the corporation, or that it was necessary, or in any degree contributed to the accomplishment of the purposes or objects for which the law authorized the corporation?

I can see no connection between the purposes to be accomplished by the creation of the corporation and its regulation touching the compensation to be exacted by its members for services to be rendered to the public authorities of Erie county and the city of Buffalo. And I have not understood the corporation or its counsel as putting the vindication of this regulation upon such a ground, and yet as we see by the well settled rules of law, unless it can be vindicated upon such ground, it cannot be sustained as a regulation or by-law binding upon the members of the corporation.

All the purposes of the corporation can be well accomplished without interfering in the least with the natural and private rights of each member to agree with the public authorities or others for such compensation for his services as he may please. The rule established by the society is unreasonable, but not that it fixed an unreasonable price for the specified services. As to that I do not know; but it is unreasonably restraining and oppressive upon its members. It interferes with their private rights.

The regulation was not only unauthorized by the law, but it is in conflict with well settled principles of law. It was the result of a combination to coerce the public authorities of Erie county and the city of Buffalo, to make a certain compensation for certain medical services, not less than a minimum sum fixed. Such a combination is, I have no doubt, unlawful at common law. It is in restraint of the right of the public authorities and the individual members of the society.

It is made the duty of the superintendents of the poor to appoint a physician for the Poor House. Act of 1851, 532. And yet if the regulation of the Medical Society of Erie County is to prevail, and is to be obeyed by the members of the society, the superintendents of the poor will not be able to procure the medical services of any one of the members of the society without paying the compensation fixed, and without any regard to the stat of health of the county poor, or the amount of services that may be required. A physician entirely competent may be willing to render the services for half the sum fixed in the tariff, and yet if he adhere to the regulation he must decline the employment. This entire regulation is in conflict with the law of the land, and cannot be sustained. It conflicts with the law and its policy, in relation to contracts and trade. The law permits and encourages great freedom in contracts and in trade, and is constantly inviting competition. The skill of the professional man is his capital in trade, and he has a right to employ it for a compensation, satisfactory to him, and thus obtain a livelihood. Ang. & Ames on Cor. 275, 3 East, 186. 1 Bl. Com. 476.

Durham *vs.* Trustees of Rochester—where a by-law was held to be unauthorized as not being prudential, and as being against the freedom of trade.

In the People *vs.* Medical Society of New York, 3 Wend. 426, it was held that an initiation fee may be demanded from physicians and surgeons on becoming members of County Medical Societies. It was held that such charge was not contrary to or inconsistent with the laws of the State, and that it was usual in most societies of this kind. It might well have been added that by section 15 of the Act of 1813 the power to charge an *admission* fee is impliedly given.

Enough has been said, I think, to show that the regulation in question was wholly unauthorized, and that it conflicts with public policy and the general law of the land. It follows that the expulsion of the relator was unjustifiable.

It seems to have been supposed, that having adopted this regulation, and having provided in the by-laws that every member who should neglect or refuse to comply with the by-laws and regulations of the society, should be expelled upon a vote of the majority of the members present, the society had the right in the way of discipline to expel a non-complying member. The rights of a member do not depend upon so frail a tenure. They are rights regulated and protected by law.

The society is not simply a voluntary association of gentlemen for social purposes or mutual improvement under rules and regulations adopted by themselves. But it is organized under the statute, and such organization is a corporation. Its powers are derived from the statute and its members have certain well defined and valuable rights. Such rights are a franchise, and expulsion is disfranchisement. I shall not stop here to show the importance of the relator's rights as a member of the society. The law has made it his duty to become a member. That his rights were important and valuable, is not denied.

Expulsion or disfranchisement is a matter of serious import, and it is important to advert to some of the rules of law relating to such questions. Disfranchisement is defined to be the taking a franchise from a man for *some reasonable cause.*

Grant on Cor. 263.—The author remarks that to entrust corporations with an arbitrary power of this kind would tend greatly to disturb the peace of corporations, and to defeat many of the objects of their institution, for it would furnish ready means to an unscrupulous majority of compassing many private and personal objects of their own by means of the corporate character. The legal causes of disfranchisement are thus stated: 1. Offences against the corporator's duty to the corporation as a member of it. 2d. Offences of a heinous, infamous character, affecting the corporator's duty as a subject, and being indictable at common law. 3d. Offences compounded of the two. It will not be necessary to notice the 2d and 3d causes. Was the relator guilty of any offence against his duty of the corporation?

It has been laid down as a rule, that such offences consist of "things done which work to the destruction of the body corporate, or to the destruction of the liberties and privileges thereof." Grant on Cor. 264. 2 Kent Com. 297 to 299. Ang & Ames on Cor. 349, 350.

This rule may be somewhat too restricted in some special cases, but it is the general and leading rule rarely departed from. If the member does acts calculated to destroy the corporation or its liberties and privileges, he may be disfranchised. He thus forfeits his right to membership.

The Commonwealth *vs.* St. Patrick's Benevolent Society, 2 Binney R. 441, is much in point in this case. In that case by a by-law *vilifying a member*, was declared to be a crime against the society, and for such crime expulsion was authorized. One of its members was convicted under this by-law and expelled.

The court, after stating and recognizing the above rules, declared the by-law void, and ordered the restoration of the expelled member. The test applied was, whether the by-law was necessary for the good government and support of the affairs of the corporation; and it was held that it was not, and that without an express power in the charter, no man can be disfranchised unless he has been guilty of some offence, which either affects the interests of good government of the corporation, or is indictable by the law of the land.

Fawcett *vs.* Charles, 13 Wend. 473, is instructive upon this question. In that case it was held that a county Medical Society had not the power to expel a member for the cause that he did not possess the requisite qualifications and obtained his admission by false pretences. The leading rules as above stated, are referred to with approbation, and it was held that such society being a body corporate has the power of expulsion as an incident to its constitution; that the power cannot be exercised without a previous conviction on indictment in a criminal court for the offence charged, except when the offence relates merely to the official or corporate character of the accused, and amounts to a breach of the condition expressly or tacitly annexed to his franchise.

By the Rev. Stat. concerning the practice of physic and surgery in this State, county medical societies are authorized to prefer specific charges against any member of gross ignorance or misconduct in his profession, or of immoral conduct or habits. These charges are to be tried by the Judges of the county court; and if they find the charges true, they are to make an order expelling the member from the society or suspending him for a limited period. R. S. 452–3.

I refer to these provisions of the statute in connection with the three general rules as above stated, touching the power of corporations to disfranchise a member for the purpose of calling attention to the nature of the charges, gross ignorance, misconduct in his profession, immoral conduct or habits, and remarking that the legislature is supposed to have understood the rules as established by the common law, and that the cases specified in the act, did not or might not come within those rules so as to authorize the societies to try the member and expel him, hence the legislature provides the remedy as specified in the statute. It did not confer the power upon the medical societies, but upon the Judges of the county court. Here is a legislative intimation, showing the extreme care taken of the rights of members of these societies, and admonishing the societies that their power of expulsion over its members is not arbitrary, but confined and carefully restricted to the causes ascertained by the common law. See matter of Newell Smith, 10 W. *ex parte*, Paine 1, Hill 665.

I have examined this case at greater length, perhaps, than was necessary. The people upon the relation of Hill against this society presenting the same and other questions arising upon the pleadings was argued very fully at the same time. In the present case it was conceded that the papers upon this motion presented fully and fairly the real question between the parties. The controversy between the society and the relator seems to have been conducted in a spirit of fairness, frankness, and courtesy — the society claiming the right of expulsion for the cause stated, and the relator controverting such right. The society is clearly in the wrong. The rights of its members to the enjoyment of their franchises are more secure than the society seems to have supposed, and the law will protect each member in the enjoyment of his rights until such rights have been legally forfeited.

1. The regulation in question was unauthorized.
2. It was unreasonable.
2. It was against public policy and the law.
4. The disfranchisement of the relator was unauthorized and illegal.

It follows that he must be restored or recognized as a member of the medical society, and permitted, without molestation, to enjoy all the rights and privileges as a member.

